# Platelet Distribution Width and Increased D-Dimer at Admission Predicts Subsequent Development of ARDS in COVID-19 Patients

**DOI:** 10.3390/pathophysiology29020019

**Published:** 2022-06-01

**Authors:** Iviana P. Yovchevska, Alexandar B. Trenovski, Maria H. Atanasova, Martin N. Georgiev, Radka K. Tafradjiiska-Hadjiolova, Simeon D. Lazarov, Plamen H. Yovchevski

**Affiliations:** 1 Department of Physiology and Pathophysiology, Medical University of Sofia, 1431 Sofia, Bulgaria; radkakir@abv.bg (R.K.T.-H.); maks2000@abv.bg (S.D.L.); 2 Clinic of Anaesthesiology and Intensive Care, Medical Institute of Ministry of Interior, 1431 Sofia, Bulgaria; atrenovski@abv.bg; 3 Clinic of Internal Medicine, Medical Institute of Ministry of Interior, 1431 Sofia, Bulgaria; mariaatanasova1994@abv.bg (M.H.A.); hristov.plmn@outlook.com (P.H.Y.); 4 Department of Microbiology, Medical Institute of Ministry of Interior, 1431 Sofia, Bulgaria; nedyalkov.martyn@gmail.com

**Keywords:** acute respiratory distress syndrome (ARDS), COVID-19, platelet distribution width, D-dimer, D-PDWf index

## Abstract

In the current pandemic of coronavirus disease (COVID-19), the identification of the patients admitted with severe infection–who are disposed to a high risk of acute respiratory distress syndrome (ARDS) development, is of a major significance for the determination of the appropriate therapeutic strategy. Laboratory records in admission were retrospectively reviewed from 493 cases of severe COVID-19 divided into two groups: Group 1 with ARDS and Group 2 without ARDS. The platelet distribution width (PDW) difference between Group 1 and Group 2 is significant–15.10 ± 2.08 fl for those who developed ARDS versus 12.94 ± 2.12 fl for those without ARDS. The sensitivity and the specificity of this parameter is lower than that of D-dimer. After grouping of the PDW values into intervals and combining them with the rate of increase in D-dimer (D-PDWf index) to form a forecasting index, a significant increase in the specificity and sensitivity of the two parameters is identified–area under the ROC curve (AUC) is 0.874 for D-PDWf index, with respective AUC for PDW 0.768 and AUC for D-dimer 0.777. Conclusion: PDW is a significant predictive parameter at admission for subsequent development of ARDS in patients, with increased significance in combination with the degree of increase in D-dimer.

## 1. Introduction

Approximately 10% of patients with COVID-19 are assumed to develop complications leading to hospital admission [1]. Thus, we identified as critical the need of predicting the severity of the development of the disease, as early as at admission.

Acute respiratory distress syndrome (ARDS) is a main cause for the severe course of the disease, respectively, its appropriate forecasting is key for clinical practice. From the pathophysiological point of view, this syndrome lies at the base of immunothrombosis [2].

Viral infections lead to the activation of the thrombocytes by several pathways. These may include direct interaction with a virus pathogen, inflammatory mediator effects from interleukin-3 and interleukin-6, exposure to viral complexes antigen-antibody [3]. Activated by these interactions, platelets become mediators of immunity [4,5].

Assessment and quantification of thrombocyte activity is typically performed via evaluation of thrombocyte indices, which can be established with a routine clinical blood test using automated hematology analyzers. PDW is an index which measures the morphology and the kinetics of the proliferation of platelets. PDW increases when high numbers of larger, younger platelets enter into circulation reflecting an increased platelet activity [6].

Assessment of D-dimers is a routine examination in the diagnostic algorithm for suspected thrombosis. D-dimer values correlate to disease severity and are trusted prognostic marker for in hospital mortality rate in COVID-19 patients. D-dimer > 1 μg/mL is one of the risk factors for death in adult COVID-19 patients [1]. The increase in D-dimer values is associated with the development of the COVID-19 associated immunothrombosis [2,7].

The main objective of this study is to evaluate the use of the PDW parameter as an indicator for the development of ARDS in severe SARS-CoV-2 infection and to answer the following questions: Is PDW appropriate to be used as a prognostic marker for the development of ARDS, in isolation or in combination with D-dimer; 2. Does PDW have a prognostic value for the mortality rates in patients with ARDS? To answer these questions, we conducted a single-center retrospective analytic case-control study covering patients with severe COVID-19.

## 2. Materials and Methods

### 2.1. Patient Selection and Data Collection

All included adult patients in the study, admitted in the period May 2020–October 2021, with PCR-confirmed (BD MAX System, Becton, NJ, USA) COVID-19 met the criteria included in the second version of the WHO–COVID-19 clinical management:

1. Moderate severity COVID-19—with manifestation of pneumonia and saturation of hemoglobin on room air during hospitalization ≥ 90%
2. Severe COVID-19—severe bilateral pneumonia plus one of the following: respiratory rate > 30 breaths per minute or SpO2 < 90% on room air [8].

The study excludes critically ill patients and those who require the provision of life-sustaining therapies such as mechanical ventilation (invasive or non-invasive) or vasopressor therapy at admission, as well as those, who at admission have PaO_2_/FiO_2_ ≤ 300 mmHg, as these are not subjects for further assessment, in line with the purpose of the study.

Patients with autoimmune disorders on immunosuppressive pathogenetic therapy, kidney and liver transplant patients were excluded from the study. No pregnant or oncology patients on radiation or hemotherapy were included.

The ARDS diagnosis is based on the criteria of Kigali Modification of the Berlin Definition: “Onset within 1 week of a known clinical insult or new or worsening respiratory symptoms; Chest imaging (radiograph, CT scan, or lung ultrasound): bilateral opacities, not fully explained by volume overload, lobar or lung collapse, or nodules; Origin of pulmonary infiltrates: respiratory failure not fully explained by cardiac failure or fluid overload. Oxygenation impairment in adults:

1. Mild ARDS: 200 mmHg < PaO_2_/FiO_2_ ≤ 300 mmHg (with PEEP or CPAP ≥ 5 cmH_2_O, or non-ventilated)
2. Moderate ARDS: 100 mmHg < PaO_2_/FiO_2_ ≤ 200 mmHg (with PEEP ≥ 5 cmH_2_O, or non-ventilated)
3. Severe ARDS: PaO_2_/FiO_2_ ≤ 100 mmHg (with PEEP ≥ 5 cmH_2_O, or non-ventilated)” [9].

These criteria are relevant in the pandemic situation, characterized by numerous people affected, some of which receive oxygen therapy with high-flow nasal cannula (HFNC). HFNC therapy is: “an oxygen supply system capable of delivering up to 100% humidified and heated oxygen at a flow rate of up to 60 L per minute, that achieves escalation of FIO2 from 21% to 100%” [10]. And despite the HFNC generated positive end-expiratory pressure (PEEP) with a closed mouth being about 1 cm of water pressure for 10 L flow, PEEP cannot be measured accurately, based on which the Berlin definition criteria are not met [10].

For the purpose of the study, we analyzed the data of 493 sequentially admitted patients meeting the investigation criteria and average age 58.0 ± 13.4 years (from 26 to 94 age). A total of two groups were formed: with ARDS—190 patients (38.5%) with average age 62.70 ± 12.66 years; and without ARDS—303 patients (61.5%) with average age 55.06 ± 13.08 years (170 females and 323 males). From female patients, 66 developed ARDS (38.8%); 124 from male (38.4%).

At a later stage, from the ARDS patients’ group, two sub-groups were identified: survivor and non-survivor. Female survivor—49 patients, non-survivor—17 patients (25.8%); male survivor—84 patients, non-survivor—40 patients (32.3%).

Patient demographical information and laboratory findings were retrospectively obtained from the patient medical history and electronic medical records from the hospital information system (HIS).

### 2.2. Laboratory Measurements

All samples were processed by the Medonic M-series automated hematology analyzer performing automated measurement of five research parameters of platelets: (PCT, PDW%, PDW, P-LCR, P-LCC) (Boule’s Diagnostic AB, Spanga, Sweden), Alinity ci-series (Abbot, Abbot Park, Illinois, USA) for clinical chemistry and immunoassay, Medica EassyBloodGas (Medica Corporation, MA, USA) for arterial blood gas test and ACL TOP 700 CTS Hemostasis analyzer (Instrumention Laboratory, Bedford, MA, USA). All measurements are taken within one hour of admission. Laboratory records from HIS were retrospectively reviewed, including white blood cell (WBC) count, hemoglobin (Hb) concentration, mean cell volume (MCV), red blood cell distribution width (RDW-CV and RDW-SD), platelets (PLT) count, granulocytes count, lymphocytes count, granulocytes/lymphocytes ratio (G/L), mean platelet volume (MPV), platelet distribution width (PDW), plateletcrit (PCT), platelet-large cell ratio (P-LCR), platelet-large cell count (P-LCC), D-dimer, creatinine, lactate dehydrogenase (LDH), C-reactive protein (CRP), ferritin, platelets/lymphocytes ratio (PLT/L), white blood cell (WBC) count/ C-reactive protein ratio (WBC/CRP).

This study was performed according to the principles laid out in the Declaration of Helsinki and was approved by the Ethics Committee of the Medical institute of Ministry of Interior (protocol 2621/9 December 2021). Every patient at admission signed an informed consent for using the medical data obtained in relation to his/her treatment for scientific purposes and education.

### 2.3. Statistical Analysis

Results were captured in a Microsoft Excel spreadsheet (Microsoft^®^, Redmond, WA, USA). Line graph and column chart in Excel were used. Statistical analyses were performed with Statistical Package for the Social Sciences^®^ software, version 28 (SPSS^®^, Chicago, IL, USA).

The data were expressed as mean ± standard deviation (SD). The differences between the groups were assessed by Student’s *t*-test. Values of *p* < 0.05 was considered statistically significant.

Receiver operating characteristics (ROC) and the corresponding area under the curve (AUC) analyses for examined parameters and indices were fulfilled to answer the identified questions. AUC 0.9 to 1 was defined as excellent accuracy, 0.8 to 0.9 as very good, 0.7 to 0.8 as good, 0.6 to 0.7 as sufficient, 0.5 to 0.6 as bad, and <0.5 as poor.

## 3. Results

The examined parameters and indices were consecutively compared in the two groups and sub-group at the time of admission. The results of two groups of patients: with ARDS and without ARDS are presented in Table 1.

Analyzing the statistically significant deviations in PDW in patients with and without ARDS, we extended the examination of the platelet distribution width by grouping it into 8 intervals:

1. PDW < 11
2. 11 ≤ PDW < 12
3. 12 ≤ PDW < 13
4. 13 ≤ PDW < 14
5. 14 ≤ PDW < 15
6. 15 ≤ PDW < 16
7. 16 ≤ PDW < 17
8. 17 ≤ PDW

**Figure 1:**
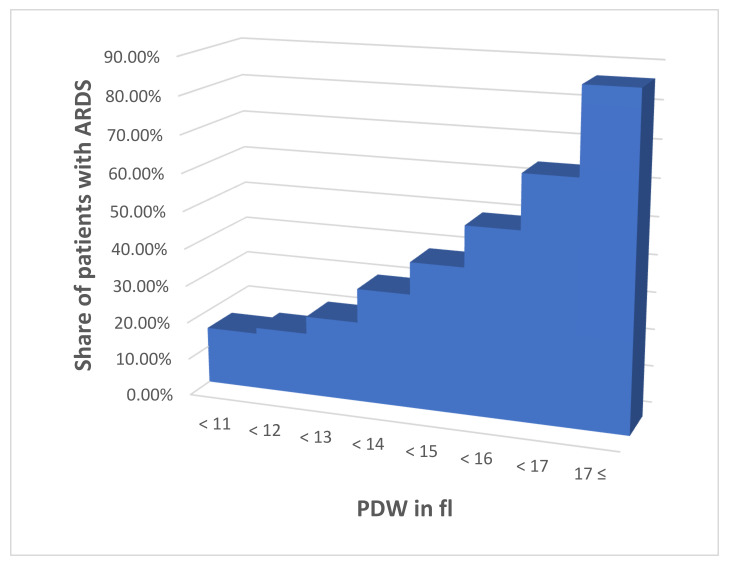
Frequency of development of ARDS in respective PDW intervals.

**Figure 2:**
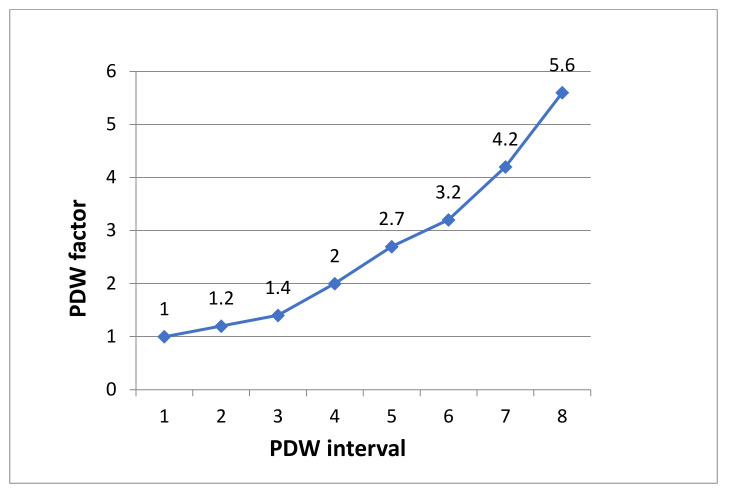
Calculated PDW factors for respective PDW intervals.

The crosstabulation of the PDW interval with the ARDS presence (Table 2) shows values of the frequency of ARDS development in the different intervals, as follows: Interval 1–15.25%, Interval 2–16.84%, Interval 3–21.52%, Interval 4–30.56%, Interval 5–39.13%, Interval 6–50.00%, Interval 7–64.39%, Interval 8–86.96% (Table 2 and Figure 1). Assessing the relation between the percentages of the ARDS development from the different intervals to that of the first one we arrived at the following PDF factor (PDWf) weight for each interval:

interval 1 1;
interval 2 1.2;
interval 3 1.4;
interval 4 2;
interval 5 2.7;
interval 6 3.2;
interval 7 4.2;
interval 8 5.6 (Table 2 and Figure 2).

**Figure 3:**
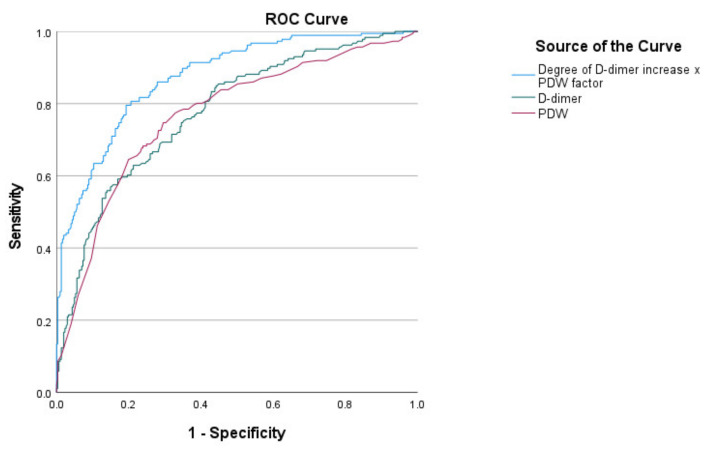

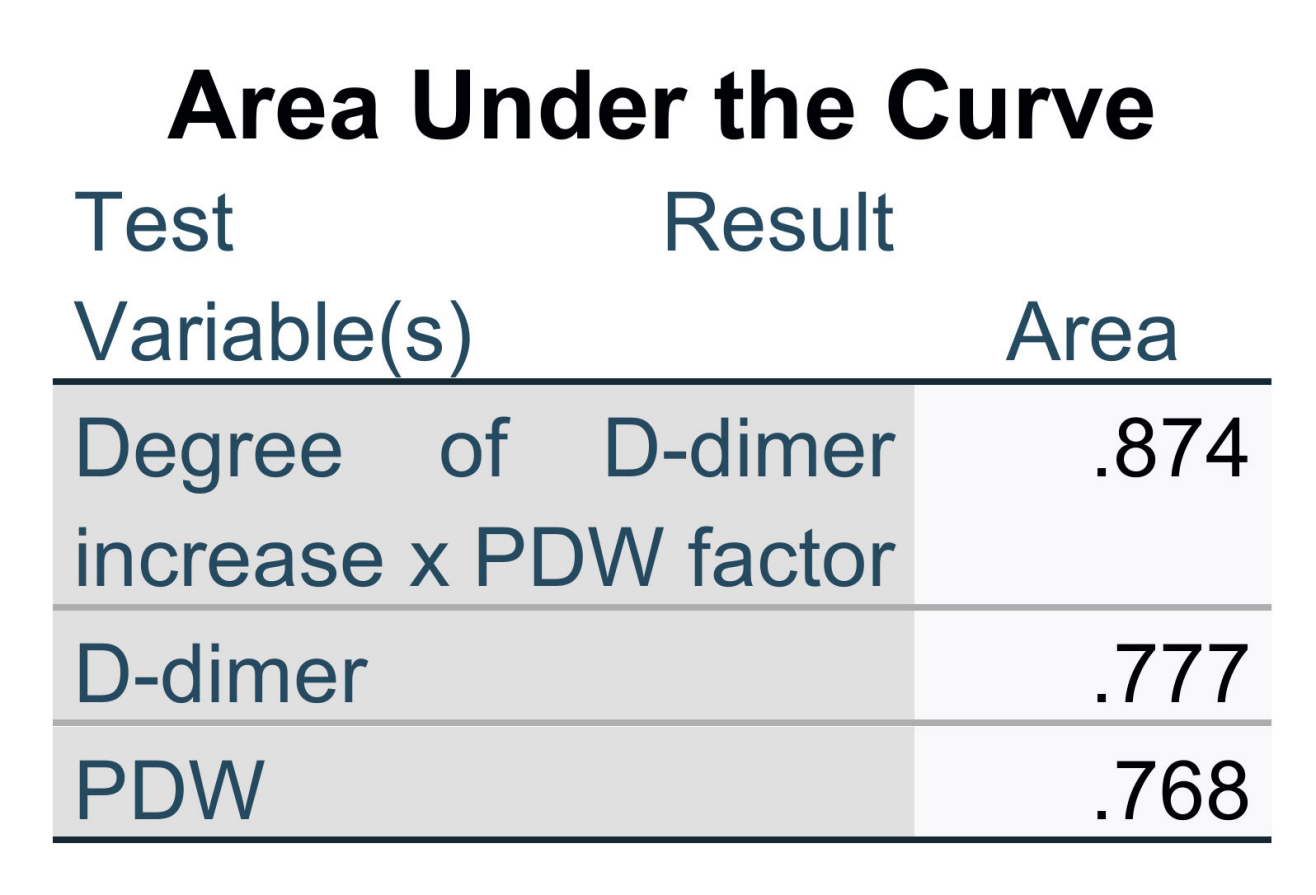
ROC curve comparing sensitivity and specificity of predicting the ARDS between PDW, D-dimer and D-PDWf.

PDWf multiplied by the degree of increase in D-dimer above the upper reference range (D-dimer/0.250) gave us a new prognostic index for the development of ARDS–D-PDWf (PDF factor × D-dimer/0.250), and specificity and sensitivity were compared with the rest of the currently applied parameters and indices. The value of the D-PDWf in patients with ARDS 7.37 ± 5.38 is statistically significantly different from its value in the patients without ARDS—2.23 ± 1.79 (*p* < 0.001). The D-PDWf indicator increases sensitivity and specificity of predicting ARDS development in comparison with PDW and D-dimer parameters in isolation (Figure 3).

When analyzing the sensitivity and the specificity of the D-PDWf index versus each of the parameters in isolation, an increased prognostic capacity of these is identified when combined in an index–area under the ROC curve for D-PDWf index: 0.874, AUC PDW: 0.768 and AUC D-dimer: 0.777. At cutoff value 3.28 for D-PDWf index the sensitivity and specificity were 79.1% and 80.7%, respectively.

**Figure 4:**
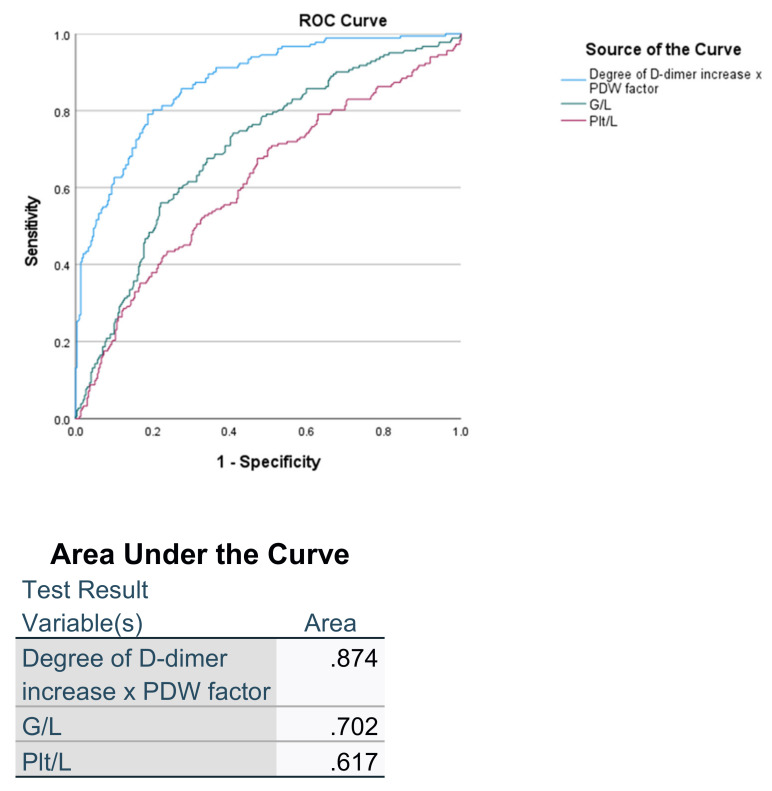
ROC curve comparing sensitivity and specificity of predicting the ARDS between D-PDWf, G/L and Plt/L.

AUC is significantly larger for the D-PDWf index, also when compared with other widely applied indices like granulocytes/lymphocytes (0.702) and platelet/lymphocytes (0.617) (Figure 4).

To answer the second question presented, we compared the parameters in the two sub-groups of patients with ARDS-survivors and non-survivors. These are shown in Table 3.

**Figure 5:**
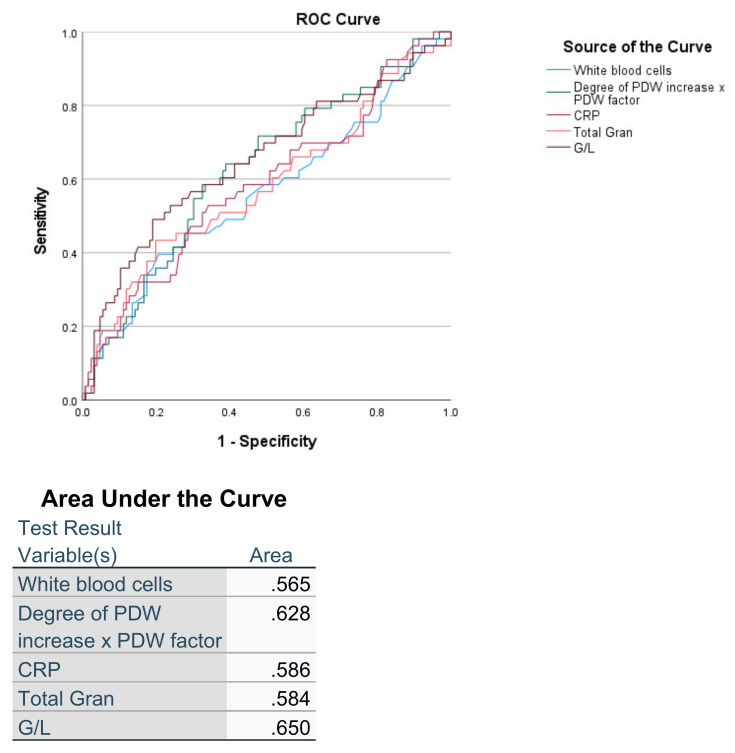
ROC curve comparing sensitivity and specificity of predicting the mortality in patients with ARDS between D-PDWf, CRP, WBC, granulocytes and G/L.

In this assessment in addition to D-PDWf, WBC, Granulocytes and their combined indicator—G/L, and CPR exhibit statistical significance, but their sensibility and specificity are low (Figure 5).

The above values of the area under the ROC curve show poor to sufficient accuracy. Based on this, in our opinion these laboratory parameters cannot be used for predicting the survival rates in patients with ARDS caused by SARS-CoV-2.

## 4. Discussion

The performed study shows, that PDW is a good prognostic indicator for the risk of ARDS development. This is relevant because the platelets are not only activated by the virus itself [11], but take part in the thrombosis [12], which is thought to be the main mechanism for development of ARDS in patients with severe COVID-19.

PDW increases during platelet activation, and this can be efficiently proven with automated hematology analyzers [13]. The more hemostatically reactive large platelets produce higher amounts of active substances from their intracellular granules, which is related to thrombotic complications [2,14]. PDW increase indicates that the larger platelets are entering the circulation as well as the presence of variations in the size of newly formed immature platelets [15].

The sensitivity of this parameter in predicting ARDS development increases significantly when combined with D-dimer, which is a proven prognostic parameter [1,2].

In assessment of the applicability of these indicators for predicting mortality or survival rates, we consider though that the use of PDW and D-PDWf, as well as the other parameters and indices analyzed in the study, for predicting the survival rates is inappropriate. Survival rates are determined by the clinical development of the disease and therapeutic measures and can be predicted by a continuous monitoring of some clinical and laboratory parameters, and not only by the parameters at admission.

In a state of pandemic, medical resources are scarce and insufficient given the numbers of those who need care. To decrease the high mortality rate (approximating 15% of the cases [16]), related to this critical illness, the use of parameters which can support the identification of high-risk patients as early as at admission is important for clinical practice.

## 5. Conclusions

PDW is significantly different between patients with ARDS and patients without ARDS. When applied in combination with D-dimer and presented as a D-PDWf index, an increase in specificity and sensibility of the prognosis is achieved. The D-PDWf index may be helpful for providing an insight when early intervention is required.

The isolated use of any of the parameters or their combination in an index does not have a sufficient prognostic value for mortality rates with the necessary specificity and sensitivity and cannot be recommended.

## Figures and Tables

**Table 1:** Comparison of age and laboratory parameters with standard deviation between Group 1 with ARDS and Group 2 without ARDS.

Parameter and Indexes	With ARDS (190)	Without ARDS (303)	*p*
Age (years)	55.06 ± 13.08	62.70 ± 12.66	<0.001
HGB (g/L)	143.13 ± 17.60	145.81 ± 14.12	=0.63
MCV (fl)	88.34 ± 6.26	86.49 ± 6.05	=0.001
RDW-CV (%)	13.82 ± 1.37	13.48 ± 0.83	<0.001
RDW-SD (fl)	45.36 ± 5.43	43.20 ± 2.60	=0.001
PLT (×10^9^/L)	229.79 ± 101.86	213.53 ± 72.13	=0.039
PCT (%)	1.559 ± 1.240	0.675 ± 0.904	<0.001
MPV (fl)	9.77 ± 1.44	8.64 ± 1.05	<0.001
PDW (fl)	15.10 ± 2.08	12.94 ± 2.12	<0.001
P-LCR (%)	29.28 ± 8.39	23.14 ± 6.88	<0.001
P-LCC (×10^9^/L)	62.99 ± 24.02	50.32 ± 16.75	<0.001
WBC (×10^9^/L)	8.47 ± 4.50	6.46 ± 2.48	<0.001
Granulocytes (×10^9^/L)	7.05 ± 4.37	4.92 ± 2.36	<0.001
Lymphocytes (×10^9^/L)	1.00 ± 0.84	1.15 ± 0.62	=0.030
D-dimer (µg/mL)	0.600 ± 0.430	0.317 ± 0.228	<0.001
Creatinine (µmol/L)	104.55 ± 66.74	90.18 ± 35.69	=0.003
LDH (IU/L)	441.80 ± 164.53	301.61 ± 128.29	<0.001
CRP (mg/L)	157.73 ± 99.41	73.15 ± 58.85	<0.001
Ferritin (ng/mL)	1,140.18 ± 1,079.55	732.44 ± 730.94	<0.001
G/L	8.99 ± 7.26	5.62 ± 4.91	<0.001
PLT/L	284.77 ± 163.08	235.35 ± 188.92	=0.003
WBC/CRP	0.082 ± 0.089	0.299 ± 0.988	=0.003

Abbreviations: HGB: Hemoglobin, MCV: Mean Cell Volume, RDW-CV and RDW-SD: red blood cell distribution width, WBC: White Blood Cells, PLT: Platelet Count, MPV: Mean Platelet Volume, PDW: Platelet distribution width, P-LCR: Platelet-Large Cell Ratio, P-LCC: Platelet-Large Cell count, PCT: Plateletcrit, LDH: lactate dehydrogenase, CRP: C-reactive protein.

**Table 2:** Crosstabulation between PDW interval and the presence of ARDS.

PDW Interval	Patients without ARDS	Patients with ARDS	Portion with ARDS → Derived Factor (% with ARDS/15.25%)
1	50	9	15.25% → 1.0
2	79	16	16.84% → 1.1
3	62	17	21.52% → 1.4
4	25	11	30.56% → 2.0
5	14	9	39.13% → 2.7
6	23	23	50.00% → 3.2
7	47	85	64.39% → 4.2
8	3	20	86.96% → 5.6

**Table 3:** Comparison of age and laboratory parameters with standard deviation between survivor and non-survivor groups.

Parameter and Indexes	Survivor (133)	Non-Survivor (57)	*p*
	61.80 ± 12.54	64.81 ± 12.79	=0.133
HGB (g/L)	143.11 ± 16.47	143.16 ± 20.15	=0.987
MCV (fl)	88.26 ± 6.64	88.53 ± 5.32	=0.787
RDW-CV	13.76 ± 1.51	13.97 ± 0.95	=0.336
RDW-SD	45.52 ± 6.01	44.94 ± 3.52	=0.641
PLT (×10^9^/L)	237.99 ± 102.11	210.65 ± 99.56	=0.090
PCT (%)	1.52 ± 1.22	1.64 ± 1.29	=0.564
MPV (fl)	9.71 ± 1.46	9.93 ± 1.39	=0.329
PDW (fl)	14.93 ± 2.16	15.48 ± 1.84	=0.095
P-LCR (%)	28.87 ± 8.10	30.20 ± 9.06	=0.417
P-LCC (×10^9^/L)	64.06 ± 23.45	60.61 ± 25.42	=0.464
WBC (×10^9^/L)	7.95 ± 3.64	9.69 ± 5.89	=0.015
Granulocytes (×10^9^/L)	6.44 ± 3.31	7.98 ± 4.66	=0.012
Lymphocytes (×10^9^/L)	1.06 ± 0.95	0.85 ± 0.48	=0.118
D-dimer (µg/mL)	0.583 ± 0.465	0.640 ± 0.333	=0.407
Creatinine (µmol/L)	99.41 ± 53.09	120.25 ± 85.74	=0.044
LDH (IU/L)	419.35 ± 134.31	496.01 ± 213.09	=0.004
CRP mg/L	147.66 ± 95.56	182.35 ± 105.10	=0.030
Ferritin (ng/mL)	1146.67 ± 1168.29	1125.42 ± 853.59	=0.904
G/L	7.67 ± 5.56	12.05 ± 9.55	<0.001
PLT/L	280.16 ± 154.72	295.46 ± 182.06	=0.559
WBC/CRP	0.086 ± 0.093	0.074 ± 0.077	=0.391
D-PDWf	6.670 ± 4.955	9.010 ± 987	=0.006

Abbreviations: HGB: hemoglobin, MCV: mean cell volume, RDW-CV and RDW-SD: red blood cell distribution width, WBC: white blood cells, PLT: platelet count, MPV: mean platelet volume, PDW: platelet distribution width, P-LCR: platelet-large cell ratio, P-LCC: platelet-large cell count, PCT: Plateletcrit, LDH: lactate dehydrogenase, CRP: C-reactive protein, D-PDWf: PDW factor × D-dimer/0.250.

## Data Availability

The datasets analyzed during the current study are available from the corresponding author on reasonable request.
